# Encouraging diversity in family engagement in research: Reflections on the development of knowledge translation tools

**DOI:** 10.1186/s40900-023-00486-7

**Published:** 2023-10-13

**Authors:** Janet W. T. Mah, Katie Nickerson

**Affiliations:** 1 BC Children’s Hospital, University of British Columbia, 4500 Oak Street, Vancouver, BC V6H 3N1 Canada; 2 Strongest Families Institute, 267 Cobequid Road, Suite 200, Lower Sackville, NS B4C 4E6 Canada

**Keywords:** Family engagement, Diversity, Partnerships, Patient-oriented research

## Abstract

**Background:**

Family engagement in research is crucial to generating relevant, impactful, and meaningful priorities and outcomes. Although there has been increased awareness and value for patient-oriented research, most patient partners in North America are from Western, educated, industrialized, rich and democratic societies. Encouraging underserviced and marginalized populations to join the partnerships is important. This project demonstrates the development of two knowledge translation tools created to encourage diversity in patient-family and researcher partnerships.

**Case study:**

Our diverse cross-Canadian team embodies the family-researcher partnership as it consists of two research personnel from non-Western origins with immigrant experiences, a parent with lived experience, and a project director. All group members have experience in the field of mental health and neurodevelopmental conditions. Four infographics were created: 3 patient-oriented ones (in English, Chinese, and Farsi) and 1 researcher-targeted one. Content for the infographics were generated to address common barriers to patient engagement identified from literature reviews, as well as key concepts discussed during the McMaster University Continuing Education Family Engagement in Research Certificate Course sponsored by CanChild & Kids Brain Health Network. Peer consultations helped to improve the infographics to be more culturally sensitive and appealing. The patient-oriented infographic presents concise bullet points about 5 main topics: (1) what is research, (2) reasons to join, (3) your role, (4) talking to researchers, and (5) how to join. The researcher-targeted infographic presents concise bullet points about 4 topics: 1) why team up with diverse patient partners, (2) ways to partner, (3) how to connect, and (4) talking to diverse partners.

**Conclusion:**

Infographics were co-designed to encourage diversity in family engagement in research. Lessons learned throughout the project include barriers encountered (e.g., team collaboration considerations, design limitations) and strategies that facilitated the project (e.g., online collaboration platforms). Future directions include translations into other languages, increased dissemination across agencies, and evaluating the effectiveness of the infographic tools.

## Abstract (plain language summary)

Family partnership in research is important because it keeps the results relevant and useful to families. Yet, most families who partner with researchers are from rich, Western societies. This means that research does not often include a variety of people and points of view. So, it is important to build research partnerships with diverse people. For our project, we created two information posters. The posters promote diversity in partnerships between researchers, patients, and families. Our own cross-Canada research team is an example of the family-researcher partnership. We share how we created content and translated it into Chinese and Farsi. We also share what we learned during the project, what we found difficult, and what was helpful. Next, we plan to translate the posters into other languages and share them with more people. We will also test the benefit of these posters.

## Background

Patient-oriented research, in which patients are engaged as active partners rather than as passive participants, is a key vision and priority among many countries across the world (e.g., Canada’s Canadian Institutes for Health Research, United States’ Patient-Centered Outcomes Research Institute, United Kingdom’s INVOLVE) [1–3]. This is important because patients have a right to be a part of every step of the research process, and their inclusion improves research relevance, study design, and dissemination [4].

To promote patient-oriented research, the Family Engagement in Research Program brings researchers and families together in an online course that is offered through McMaster University and sponsored by CanChild & Kids Brain Health Network [5]. Course participants include a mix of researchers (e.g., graduate students, research coordinators, investigators, clinician-researchers) and families (e.g., parents, siblings, grandparents) who have an interest in child neurodevelopmental research. Over ten weeks, readings and group discussions help learners gain a better understanding of the importance of family engagement in research, how to engage families throughout the research process, barriers/facilitators to engagement, ethics surrounding engagement, and tools and resources to support and evaluate engagement.

As part of the course, participants complete a group project to create a knowledge translation (KT) tool about patient partnerships in research. There have been many knowledge translation tools developed by course participants on a variety of topics, such as websites to introduce families to research engagement, guidelines on starting and maintaining partnerships, and blogs and social media platforms to engage youth and families [6]. However, we noticed that none has addressed cultural diversity. Many research participants and partners are those from Western, educated, industrialized, rich, and democratic (WEIRD) societies [7]. Research should reflect the diversity of human society [8], so we wanted to encourage diversity in researcher-family partnerships, given the Canadian multicultural mosaic and how families from racialized minorities are under-represented in the fields of mental health and disability [9].

## Our project

Thus, we chose to develop knowledge translation tools focused on enhancing immigrant families’ understanding of research and the importance of their engagement in meaningful research partnerships. At the same time, we acknowledged that we also needed to target researchers to encourage them to authentically include immigrant families in research, not simply as data points, but as partners throughout the research process. We hoped that these KT tools would enable new Canadian and under-serviced communities to benefit from interactions with healthcare and research professionals in order to help build a diverse, inclusive, equitable, and accessible research community. Project outcomes arising from diverse family-research partnerships would in turn result in enhanced applications to culturally-appropriate health services.

### Team

Our team embodied the family-researcher partnership as it consisted of two clinical researchers, a project director in the field of mental health and neurodevelopmental conditions, and a parent with lived experience. We also demonstrated diversity in ethnic/cultural backgrounds (e.g., two members were from non-Western origins with immigrant experiences), interdisciplinary professions (e.g., psychology, medicine, editor), and geographical locations (i.e., spanning cross-Canada from British Columbia, Alberta, Nova Scotia, and Ontario).

### Project development

All team members shared our cumulative experiences and perspectives related to culture, stigma, and obstacles to healthcare and research engagement. The first author conducted a literature search to explore existing knowledge about addressing barriers to research engagement and service use by racialized populations. Although no published articles were found at the time specifically about barriers to patient engagement in research among racialized populations, we chose to focus on one article that highlighted barriers to research engagement identified from the public's view [10], and one report on barriers to service use by racialized populations [11].

We chose to develop the KT tools in the form of infographics. We thought that this format would be visually engaging, with simple text that accommodates for varying literacy and comprehension levels, and offer ease of distribution across settings. We decided to create two infographics to encourage cultural diversity in research partnerships: one targeting families from racialized groups, and one targeting researchers. The second author led the design of the infographics using Canva [12], an online design and publishing program.

### Translations

Given the cultural composition of our team, we decided to translate the family infographic from English into Chinese (traditional) and Farsi. We developed the content first in English, which was then translated into the other language by someone with linguistic and cultural competence in English and Chinese or Farsi. The translations were then reviewed by independent individuals from Chinese or Iranian communities for linguistic and cultural appropriateness.

### Feedback

We sought consultations from peers, course instructors, and community partners for feedback regarding design, language, and content of the four infographics developed (family resource in English, Chinese and Farsi, and researcher resource in English). Six peers, five instructors, and three community partners from various cultural, professional, and geographical backgrounds provided written feedback identifying:

- The importance of the topic and appreciation for the commitment to greater diversity in research,
- Good use of engaging visual colours, fonts, and spacing, with precise and succinct content, and
- Suggestions for minor edits in wording or content for clarity and inclusiveness.

Most of the recommendations for changes were incorporated while keeping the infographics simple and engaging. 

### Tool for families

**Fig. 1:**
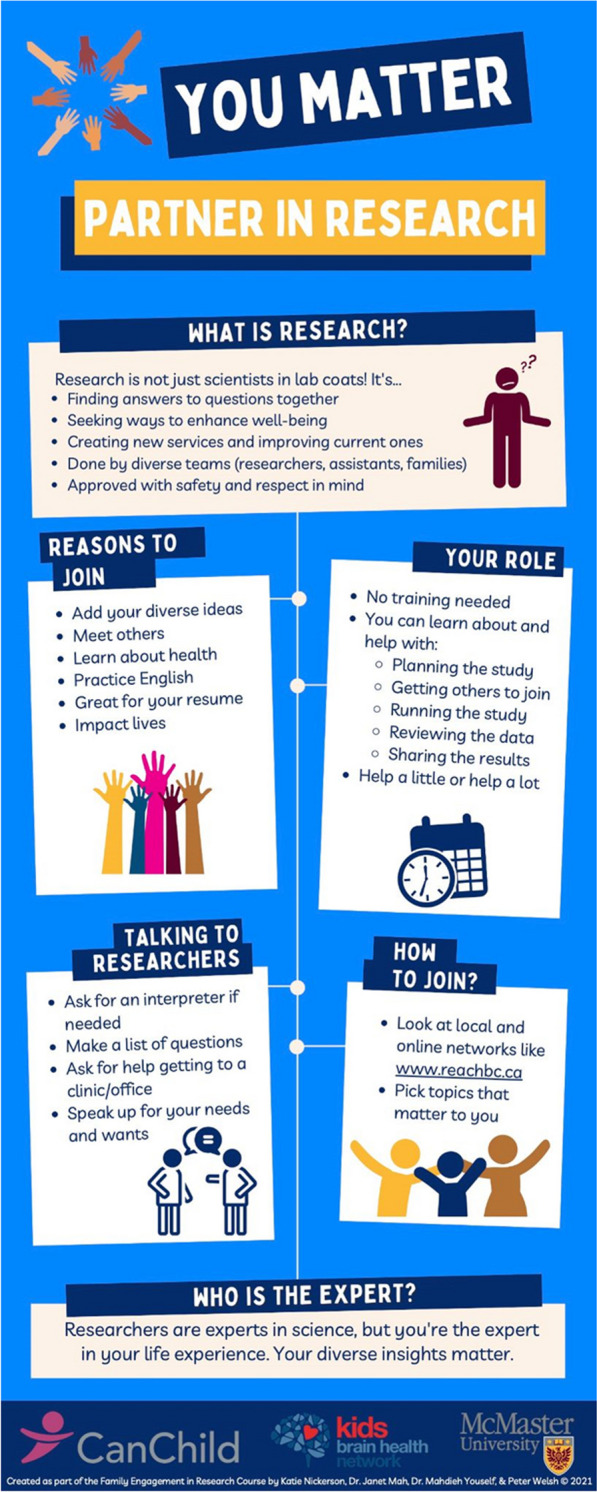
Infographic for Families in English

For the family-focused tool (see Fig. 1 for English version; see appendix for Chinese and Farsi versions), we included basics about what research is, benefits to engaging in research, roles and commitment, and tips for connecting with researchers. In particular, we sought to address six barriers to research engagement identified from the public's view, which may be more representative of the underserviced racialized populations whose voices may not be captured in literature focused on patient engagement [10] – see Table 1.

**Table 1:** Infographic tool addresses barriers for family engagement in research

Identified barrier	How the infographic addresses It
lack of relevance	Add your diverse ideas
lack of impact	Research creates new services and improves current ones
lack of trust	Research is approved with safety and respect in mind
lack of legitimacy	Your diverse insights matter
lack of knowledge	No training needed
	You’re the expert in life experience
lack of time and financial resources	Help a little or a lot
	Ask for help. Speak up for your needs and wants

### Tool for researchers

**Fig. 2:**
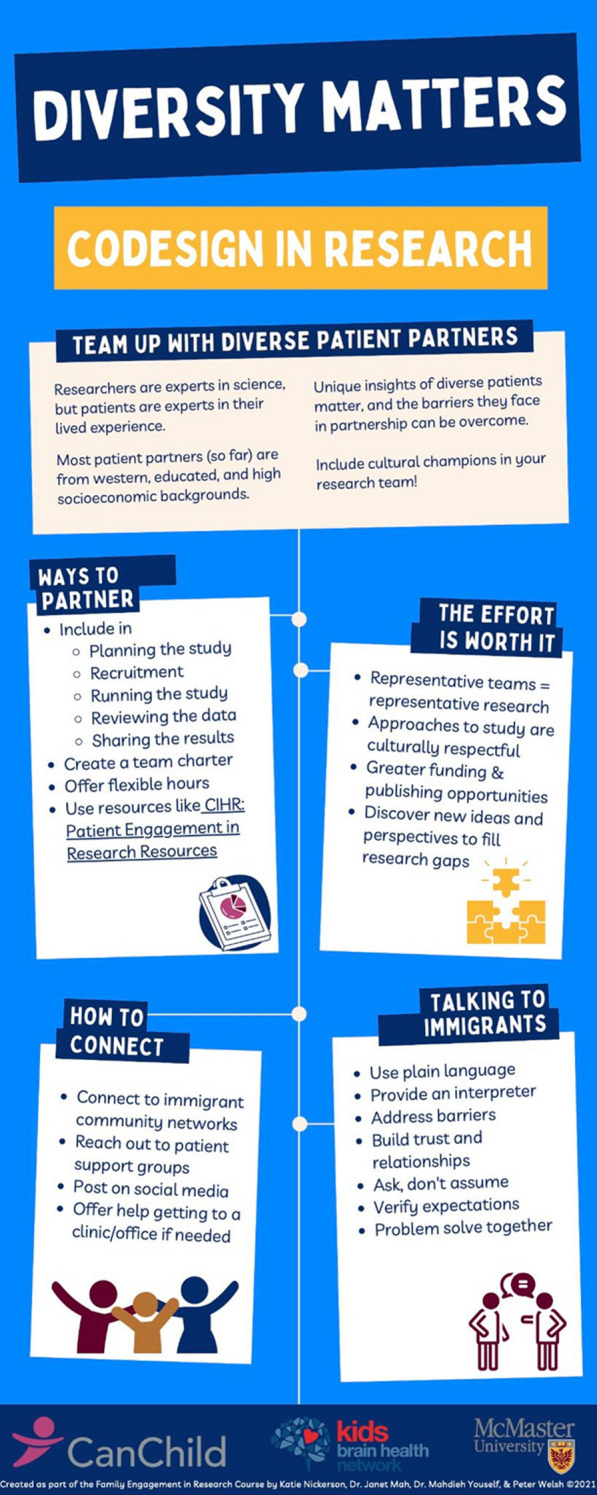
Researcher Infographic

The researcher-focused tool (see Fig. 2) highlights reasons to include diverse patient partners, suggested ways to partner, and tips on connecting and communicating. Furthermore, we also sought to address six common barriers to service use by racialized populations identified by the Mental Health Commission of Canada [11] – see Table 2.

**Table:** Infographic tool addresses barriers for the researcher to consider

Identified barrier	How the infographic addresses It
Service accessibility	Offer flexible hours
	Connect to community networks/support groups/social media
Patient-provider interaction	Problem solve together
	Include cultural champions in your research team
Circumstantial challenges	Offer help getting there. Address barriers
Language	Use plain language. Provide an interpreter
Stigma	Build trust and relationships
Fear	Verify expectations. Ask, don’t assume

## Discussion

Our project demonstrates the creation of knowledge translation tools specifically aimed at encouraging patient-oriented research, particularly among diverse cultural populations. The project was developed by a multi-disciplinary team that included a family partner with lived experience. Through this collaboration, and with consideration of literature reviews and course content about key factors to consider for family engagement in research, four infographics were developed to encourage cultural diversity in family and researcher partnerships: one targeting researchers, one targeting families offered in three languages – English, Chinese, and Farsi.

### Barriers encountered

We faced a few barriers during the development of this project. First, team diversity was both a strength and a challenge. Although our different professional and cultural backgrounds helped to enhance our project, it also contributed to differences in communication and working styles. For instance, lots of time (e.g., weeks for initial introduction and orientation) was needed for each team member to share their ideas and come to consensus about the project direction, content, and process. In addition, because we were based in different locations across Canada, it was tricky to find common meeting times across time zones while also working around scheduling conflicts from our professional day jobs. This was particularly important to consider for our family partner who did not have paid time to commit to this project. These are considerations that family-researcher partnerships also encounter.

Second, although infographics are advantageous for visual engagement, there are also limitations of available space for content, finding inclusive & meaningful images that are culturally sensitive, and selecting the appropriate words that would suit language and comprehension levels to accommodate English-as-a-second-language competencies. None of the team members had professional experience or training in design, and the project was unfunded, so access to design expertise and resources was also a constraint.

### Lessons learned

Through this project, we learned that it’s helpful to define roles and responsibilities early. In particular, identifying skills and interests of each team member can help to match up individuals with tasks and duties required. In addition, we think it may have been useful to designate an individual to be the project coordinator to keep the team accountable in following through with tasks. In a family-researcher partnership, we think having an independent project coordinator could also help bridge expectations and communication to reduce power imbalances and foster stronger alliances.

Second, we benefited from using technology for synchronous and asynchronous collaboration. For this project, we used Canva to design the infographics, as well as Google Docs and Slides for content and presentations. These online programs allow each team member to edit the documents independently as well as simultaneously.

Finally, yet importantly, our team relied on a handy motto: GELMO, meaning “good enough, let’s move on!” It was easy to go in circles when collaborating with others as we tried to respectfully consider the varied opinions and ideas, weighing the pros and cons of each. GELMO allowed us to be more efficient with our time and enhanced our effectiveness as a team.

### Future directions

The developed infographics are currently posted on CanChild. We have presented our work at a provincial conference on patient-oriented research in British Columbia, Canada. There has been interest expressed from others in additional translations into other languages besides Chinese and Farsi. Further dissemination of the KT infographics can be done through research networks, community agencies, and cultural organisations. These traditional recruitment methods of advertising can also be supplemented with sampling frameworks to recruit hard-to-reach populations, such as ethnographically mapping popular venues frequented by racialized communities, and/or peer recruitment in which original participants or champions from racialized groups connect with other potential participants [13]. It would also be useful to evaluate the effectiveness of the KT tools in increasing family-researcher partnerships among culturally diverse populations before and after its dissemination.

## Conclusions

Two infographics were created to encourage diversity in family engagement in research. One targeting families from immigrant backgrounds to address common barriers (in English, Chinese, and Farsi); one targeting researchers about the benefits of teaming up with diverse family partners. Lessons learned include barriers encountered (e.g., team collaboration considerations, design limitations) and strategies that facilitated the project (e.g., online collaboration platforms). These infographics can be disseminated broadly as KT tools so the voices of diverse groups can be included in family-research partnerships for more meaningful and impactful project priorities and outcomes.

## Data Availability

Not applicable.

## Abbreviations

GELMO: Good enough, let’s move on!
KT: Knowledge translation
WEIRD: Western, educated, industrialized, rich and democratic

## References

[1] INVOLVE. 2020. [cited 2020 Jan 17]. Available from: https://www.invo.org.uk.

[2] PCORI. 2020. [cited 2020 Jan 17]. Available from: https://www.pcori.org.

[3] SPOR. 2020. [cited 2020 Jan 17]. Available from: https://cihr-irsc.gc.ca/e/41204.html.

[4] Duffett L (2017). Patient engagement: what partnering with patients in research is all about. Thromb Res.

[5] CanChild centre for childhood disability research. family engagement in research course. [Internet]. Available from: https://www.canchild.ca/en/research-in-practice/family-engagement-program/fer-course.

[6] CanChild centre for childhood disability research. Student projects. [Internet]. Available from: https://kidsbrainhealth.ca/training/training-careers-opp/family-engagement-in-research-course/ferc-student-projects/

[7] Henrich J, Heine S, Norenzayan A (2010). Beyond WEIRD: towards a broad-based behavioral science. Behav Brain Sci.

[8] Research governance framework for health and social care, 2nd edition. Department of Health; 2005; Apr.

[9] Le Cook B, Trinh NH, Li Z, Hou SS, Progovac AM (2017). Trends in racial-ethnic disparities in access to mental health care, 2004–2012. Psychiatr Serv.

[10] Dreyer M, Kosow H, Bauer A (2017). Public engagement with research: citizens’ views on motivations, barriers and support. Res All.

[11] Mental health commission of Canada. The case for diversity: building the case to improve mental health services for immigrant, refugee, ethno-cultural and racialized populations. Ottawa, ON: Mental Health Commission of Canada; 2016.

[12] Canva. [Internet]. Canva. [cited 2023 Aug 11]. Available from http://www.canva.com.

[13] Golenya R, Chloros GD, Panteli M, Giannoudis PV (2021). How to improve diversity in patient and public involvement. Br J Hosp Med.

